# Predictors of bronchopulmonary dysplasia occurrence and severity in extremely preterm infants

**DOI:** 10.3389/fped.2025.1629279

**Published:** 2025-08-12

**Authors:** Xuejing Liu, Wanxian Zhang, Fangrui Ding

**Affiliations:** ^1^ Department of Neonatology, Tianjin Central Hospital of Obstetrics and Gynecology, Tianjin, China; ^2^ Department of Neonatology, Nankai University Affiliated Maternity Hospital, Tianjin, China; ^3^ Tianjin Key Laboratory of Human Development and Reproductive Regulation, Tianjin Central Hospital of Obstetrics and Gynecology, Tianjin, China

**Keywords:** extremely premature infants, bronchopulmonary dysplasia, invasive mechanical ventilation, transfusion, fluid balance, antibiotics, patent ductus arteriosus

## Abstract

**Background:**

Bronchopulmonary dysplasia (BPD) is a common complication in extremely preterm infants (EPIs), and there is currently a lack of effective preventive strategies. Identifying risk factors may facilitate early interventions and improve outcomes.

**Objective:**

To investigate risk factors for the occurrence and severity of BPD in EPIs and inform potential prevention strategies.

**Methods:**

We conducted a retrospective analysis of medical records from EPIs admitted to the neonatal intensive care unit at Tianjin Central Hospital of Obstetrics and Gynecology between 2012 and 2024. BPD was diagnosed according to the 2018 revised criteria established by the National Institute of Child Health and Human Development. Multivariable logistic regression was used to identify independent risk factors.

**Results:**

Among 468 EPIs, 136 (29.1%) developed BPD (mild: 14.1%, moderate: 7.1%, severe: 7.9%). Independent risk factors for BPD included prolonged invasive mechanical ventilation (IMV, OR = 1.10, 95% CI 1.03–1.17), frequent red blood cell transfusions (RBCTs, OR = 1.61, 95% CI 1.30–2.01), extended antibiotic exposure (OR = 1.03, 95% CI 1.01–1.06), and hemodynamically significant patent ductus arteriosus (hsPDA, OR = 2.27, 95% CI 1.22–4.20). Prolonged IMV (OR = 1.16, 95% CI 1.06–1.27) and higher fluid balance (FB) on postnatal day 7 (OR = 1.19, 95% CI 1.05–1.34) were independent risk factors for moderate-to-severe BPD, while higher birth weight (OR = 0.99, 95% CI 0.988–0.998) was found to be a protective factor. Whole blood transfusion was associated with an increased risk of BPD (OR = 4.48, 95% CI 1.92–10.43) and moderate-to-severe BPD (OR = 4.81, 95% CI 1.24–18.63) compared to packed RBCTs. In predicting moderate-to-severe BPD, the duration of IMV (cut-off: 6.5 days) and FB on postnatal day 7 (cut-off: −7.2) demonstrated significant predictive value.

**Conclusions:**

In conclusion, the occurrence and severity of BPD in EPIs are influenced by prolonged IMV, frequent RBCTs, fluid overload, excessive antibiotic exposure, and hsPDA. Early interventions targeting modifiable factors, such as reducing IMV duration, maintaining an appropriate negative FB on postnatal day 7, and optimizing transfusion protocols, are critical to prevent moderate-to-severe BPD.

## 1 Introduction

Bronchopulmonary dysplasia (BPD) remains one of the most prevalent and serious complications affecting extremely preterm infants (EPIs), defined as those born at less than 28 weeks of gestational age (GA). Over the past few decades, advances in neonatal care particularly in high-resource settings have led to remarkable improvements in the survival of EPIs. However, this progress has been accompanied by a concerning rise in the incidence of BPD (1–3). BPD is associated with significant long-term morbidity, including cardiorespiratory dysfunction, impaired growth, and neurodevelopmental delays (4–6), with moderate-to-severe BPD imposing the highest burden. These sequelae often extend into adulthood, imposing substantial emotional and financial burdens on affected families and healthcare systems.

Numerous studies have investigated risk factors for BPD, however, the majority have focused on preterm infants with a GA <32 weeks and birth weight (BW) <1,500 g, particularly in the Chinese population. However, given that EPIs are at the highest risk for BPD, this study focuses exclusively on EPIs to evaluate the risk factors for BPD. By doing so, we aim to provide evidence-based insights that can guide targeted strategies for the prevention and management of BPD in this vulnerable population.

## 2 Materials and methods

### 2.1 Study population

This retrospective study included preterm infants with a GA <28 weeks admitted to the neonatal intensive care unit (NICU) at Tianjin Central Hospital of Obstetrics and Gynecology within 24 h of birth from January 2012 to December 2024. Exclusion criteria were: (1) length of hospital stay <14 days; (2) inter-hospital transfer; (3) congenital anomalies (e.g., complex congenital heart disease, chromosomal abnormalities, etc.); (4) death prior to 36 weeks postmenstrual age (PMA) due to non-persistent parenchymal lung disease and respiratory failure.

### 2.2 Data collection

General clinical data included: (1) Demographic factors: gestational age (GA), birth weight (BW), sex, multiple pregnancy (vs. singleton pregnancy), mode of delivery (cesarean vs. vaginal), and 5-minute Apgar score. (2) Maternal factors: maternal age, antenatal corticosteroid use, premature rupture of membranes (PROM), gestational diabetes mellitus (GDM), and hypertensive disorders of pregnancy (HDP). (3) Complications: pulmonary hemorrhage, sepsis (including early-onset and late-onset sepsis), necrotizing enterocolitis (NEC), hemodynamically significant patent ductus arteriosus (hsPDA), extrauterine growth restriction (EUGR), intraventricular hemorrhage (IVH) (grade 3/4 IVH vs. no IVH or grade 1/2 IVH), and retinopathy of prematurity (ROP). (4) Treatment-related factors: use of surfactant, ventilation modalities: invasive mechanical ventilation [IMV; categorized into high-frequency oscillatory ventilation [HFOV], conventional mechanical ventilation [CMV], and combined HFOV + CMV therapy] and non-invasive ventilation (NIV), red blood cell transfusions (RBCTs): frequency and product type (whole blood vs. packed red blood cells), duration of antibiotic therapy, duration of postnatal corticosteroid use, feeding method (breastfeeding vs. formula), fluid balance (FB) on postnatal days 3 and 7, achievement of caloric intake targets (>100 kcal/kg/day by week 1, >120 kcal/kg/day by week 4). (5) Outcomes: death or survival. Due to limited use of noninvasive positive pressure ventilation (NIPPV) and universal transition to continuous positive airway pressure (CPAP), NIPPV cases were classified as CPAP. All high-flow nasal cannula (HFNC) recipients had prior CPAP exposure and were designated as the CPAP + HFNC combination group. Data collection for variables potentially associated with BPD risk was completed prior to 36 weeks PMA.

Standardized interventions: Initial delivery room stabilization utilized a T-resuscitator, with CPAP as the primary intervention based on spontaneous breathing and heart rate assessment. All EPIs received prophylactic caffeine citrate (20 mg/kg loading dose, followed by 5–10 mg/kg/day maintenance).

### 2.3 Diagnostic criteria

#### 2.3.1 BPD

The diagnosis of BPD was based on the 2018 criteria established by the National Institute of Child Health and Human Development (NICHD) (7). Infants with a GA <32 weeks were diagnosed with BPD if they exhibited persistent radiographic evidence of parenchymal lung disease and required respiratory support for at least three consecutive days at 36 weeks PMA to maintain arterial oxygen saturation between 90% and 95%. BPD severity was classified into grades I, II, III, and IIIA based on the level of respiratory support and fractional inspired oxygen (FiO₂), as detailed in Table 1. For analysis, grade I was categorized as mild BPD, while grades II, III, and IIIA were grouped as moderate-to-severe BPD.

**Table 1:** BPD grades of the criteria modified by the NICHD in 2018.

Grades	IMV^a^	N-CPAP, NIPPV, or NC ≥3 L/min	NC 1–2 L/min	Hood O_2_	NC <1 L/min
I	-	21%	22%–29%	22%–29%	22%–70%
Ⅱ	21%	22%–29%	≥ 30%	≥ 30%	> 70%
Ⅲ	> 21%	≥ 30%	-	-	-
ⅢA	Early death (between 14 days of postnatal age and 36 weeks) owing to persistent parenchymal lung disease and respiratory failure that cannot be attributable to other neonatal morbidities.				

^a^ Infants requiring mechanical ventilation due to primary airway disease or central respiratory failure were excluded. Percentages in the table represent fractional inspired oxygen. IMV, invasive mechanical ventilation; N-CPAP, nasal continuous positive airway pressure; NIPPV, noninvasive positive pressure ventilation; NC, nasal cannula.

#### 2.3.2 Other diagnostic criteria

FB was calculated by comparing daily weight with BW, in accordance with a previous study (8), using the following formula: FB = [(Daily Weight – BW)/BW] × 100. We calculated the FB on postnatal days 3 and 7. Hemodynamically significant patent ductus arteriosus (hsPDA) was defined using a combination of clinical and echocardiographic criteria (9). Clinical signs included the presence of a heart murmur, tachycardia, bounding pulses, hyperdynamic precordial impulse, wide pulse pressure, or worsening respiratory status. Echocardiographic and Doppler findings required for diagnosis included: (1) demonstration of left-to-right shunt; (2) left atrial-to-aortic root ratio >1.3; (3) ductal diameter >1.5 mm; and (4) disturbed diastolic flow in the main pulmonary artery. NEC was defined as Bell's stage II or higher (10). EUGR was identified if an infant's weight at 36 weeks PMA or at discharge was below the 10th percentile on the Fenton 2013 growth curves (11).

### 2.4 Statistical analysis

Data were analyzed using SPSS 29.0. Categorical variables were presented as *n* (%) and compared with Chi-square tests or continuity-corrected Chi-square tests. Bonferroni correction was applied for multiple comparisons. Normally distributed continuous variables were described using mean ± standard deviation (*SD*) and analyzed with independent *t*-tests. Non-normally distributed continuous variables were expressed as median (interquartile ranges, *IQR*) and compared using Mann–Whitney *U*-tests. Logistic regression was performed to identify risk factors for BPD and moderate-severe BPD. Variables considered clinically important and those for which a statistically significant difference (*p* < 0.10) was observed in the univariate analysis were included in the multivariate models, using Akaike information criterion (AIC) as the selection criterion in a stepwise forward selection strategy. The goodness of fit of the logistic regression models was assessed using the Hosmer-Lemeshow test and the Omnibus test. Predictive accuracy was evaluated using Cox and Snell's *R*^2^ and Nagelkerke's *R*^2^ (to quantify explained variance) and receiver operating characteristic (ROC) curves with area under the curve (AUC). Statistical significance was defined as *p* < 0.05.

## 3 Results

### 3.1 General characteristics

A total of 649 EPIs were admitted during the study period. After applying the exclusion criteria, 181 infants were excluded, leaving 468 infants for final analysis. Among them, 7 infants (1.5%) had a GA <24 weeks, 40 (8.5%) were 24 weeks, 72 (15.4%) were 25 weeks, 143 (30.6%) were 26 weeks, and 206 (44.0%) were 27 weeks, with a median GA of 26.6 weeks (*IQR*: 25.6–27.2). Regarding BW, 52 infants (11.1%) weighed <750 g, 261 (55.8%) weighed 750–1,000 g, and 155 (33.1%) weighed ≥1,000 g, with a median BW of 930 g (*IQR*: 820–1,030 g). There were 259 males (55.3%) and 209 females (44.7%).

### 3.2 Incidence and clinical characteristics of BPD

A total of 136 infants (29.1%) developed BPD, with 66 cases (14.1%) classified as grade I (mild), 33 (7.1%) as grade II, 20 (4.3%) as grade III, and 17 (3.6%) as grade IIIA. For analytical purposes, grades II, III, and IIIA were combined into a moderate-to-severe BPD category. Compared to 2012–2019, the 2020–2024 cohort exhibited stable survival rates (69.0% vs. 69.7%, *p* = 0.851) but demonstrated significantly elevated incidences of overall BPD (34.5% vs. 24.2%, *p* = 0.014), moderate-to-severe BPD (19.1% vs. 11.3%, *p* = 0.018), and a higher proportion of infants with GA <26 weeks (30.5% vs. 21.0%, *p* = 0.019). GA demonstrated a strong inverse relationship with both BPD and moderate-to-severe BPD incidence. Infants with GA <26 weeks, especially those ≤24 weeks, showed significantly higher BPD incidence (48.6–63.8%) and moderate-to-severe BPD rates (57.1–76.7%) compared to 26–27 week cohorts. Details are presented in Tables 2, 3.

**Table 2:** Comparison of BPD incidence, gestational age distribution, and survival outcomes in EPIs: 2012–2019 vs. 2020–2024.

Variable	2012–2019	2020–2024	*p*
Any BPD, *n/N* (%)	60/248 (24.2)	76/220 (34.5)	0.014
Mild BPD, *n/N* (%)	32/248 (12.9)	34/220 (15.5)	0.429
Moderate-to-Severe BPD, *n/N* (%)	28/248 (11.3)	42/220 (19.1)	0.018
GA <26w, *n/N* (%)	52/248 (21.0)	67/220 (30.5)	0.019
Survival, *n/N* (%)^a^	232/333 (69.7)	218/316 (69.0)	0.851

^a^ Survival rate denominators include all eligible infants, including those not enrolled in the study. BPD, bronchopulmonary dysplasia; GA, gestational age.

**Table 3:** Comparison of BPD and moderate-to-severe BPD by clinical subgroups.

Variable	*n*	BPD	*p*	*n*	Moderate-to-Severe BPD	*p*
GA, *n* (%)						
≤24w	47	30 (63.8)^a^	<0.001	30	23 (76.7)^a^	0.002
25w	72	35 (48.6)^a^		35	20 (57.1)^a^^,^^b^	
26w	143	36 (25.2)^b^		36	16 (44.4)^b^	
27w	206	35 (17.0)^b^		35	11 (31.4)^b^	
IMV, *n* (%)						
HFOV	18	9 (50.0)	0.367	9	6 (66.7)	0.135
CMV	54	35 (64.8)		35	21 (60.0)	
HFOV + CMV	36	25 (69.4)		25	21 (84.0)	
IMV, median (*IQR*), days						
HFOV	18	15 (3–23)	0.148	9	19 (14–26)	0.700
CMV	54	10 (6–20)		35	16 (9–35)	
HFOV + CMV	36	19 (9–26)		25	21 (13–32)	
NIV, *n* (%)						
CPAP	405	79 (19.5)	<0.001	79	28 (35.4)	<0.001
CPAP + HFNC	51	48 (94.1)		48	33 (68.8)	
NIV, median (*IQR*), days						
CPAP	405	56 (33–73)	<0.001	79	56 (17–77)	0.001
CPAP + HFNC	51	77 (61–92)		48	85 (64–94)	
RBCTs, *n* (%)						
Packed RBCTs	169	49 (29.0)	<0.001	49	19 (38.8)	0.008
Whole blood	103	64 (62.1)		64	41 (64.1)	

BPD, bronchopulmonary dysplasia; GA, gestational age; IMV, invasive mechanical ventilation; HFOV, high-frequency oscillation ventilation; CMV conventional mechanical ventilation; NIV, non-invasive ventilation; CPAP, continuous positive airway pressure; HFNC, high-flow nasal cannula; RBCTs, red blood cell transfusions.

^a,b^ Pairwise comparisons between gestational age groups were performed using Bonferroni correction. Groups sharing the same superscript letter indicate no statistically significant difference; differing letters denote significant differences.

Compared to the non-BPD group, infants with BPD exhibited significantly lower GA and BW, lower rate of NIV, along with elevated rates of 5 min Apgar scores <7, mortality, need for and duration of IMV, transfusion frequency and number of RBCTs, duration of antibiotic use, and postnatal corticosteroid exposure. Additionally, the BPD group had higher incidences of pulmonary hemorrhage, sepsis, NEC, and hsPDA. In contrast, the BPD group had a lower proportion of infants achieving caloric intake targets: >100 kcal/kg/day by the end of week 1 and >120 kcal/kg/day by the end of week 4 of life (all *p* < 0.05). No significant differences were observed between the groups in terms of sex, multifetal pregnancy, mode of delivery, maternal age, maternal complications, antenatal corticosteroid use, feeding methods, use of surfactant, FB on postnatal days 3 and 7, EUGR, grade 3/4 IVH, or ROP (all *p* > 0.05). When comparing the moderate-to-severe BPD group to the mild BPD group, the former group had significantly lower GA and BW, higher mortality rates, lower rate of NIV, greater need for and duration of IMV, increased frequency and number of RBCTs, higher FB on postnatal days 3 and 7, and higher incidence of pulmonary hemorrhage. The moderate-to-severe BPD group also had lower rates of breastfeeding, lower incidences of ROP, and a lower proportion of infants achieving caloric intake target of >120 kcal/kg/day by the end of week 4 of life (all *p* < 0.05). See Table 4 for additional details.

**Table 4:** Demographic and clinical characteristics of the study subjects.

Variable	Non-BPD	BPD	*p*	Mild BPD	Moderate-to-Severe BPD	*p*
	*n* = 332	*n* = 136		*n* = 66	*n* = 70	
GA, median (*IQR*), week	27.0 (26.2–27.3)	26.1 (25.1–27.0)	<0.001	26.5 (25.5–27.2)	25.4 (24.5–26.5)	<0.001
BW, median (*IQR*), g	950 (860–1,058)	845 (750–980)	<0.001	915 (818–1,068)	785 (698–880)	<0.001
Death, *n* (%)	2 (0.6)	25 (18.4)	<0.001	1 (1.5)	24 (34.3)	<0.001
Male, *n* (%)	181 (54.5)	78 (57.4)	0.575	42 (63.6)	36 (51.4)	0.150
Multiple pregnancy, *n* (%)	106 (31.9)	53 (39.0)	0.144	30 (45.5)	23 (32.9)	0.132
Cesarean delivery, *n* (%)	56 (16.9)	23 (16.9)	0.991	11 (16.7)	12 (17.1)	0.941
5 min Apgar scores ≤7, *n* (%)	94 (28.3)	54 (39.7)	0.016	27 (40.9)	27 (38.6)	0.781
Maternal age, median (*IQR*), year	32 (29–36)	32 (29–35)	0.511	31 (29–34)	32 (29–35)	0.218
Antenatal corticosteroids, *n* (%)	234 (70.5)	94 (69.1)	0.770	42 (63.6)	52 (74.3)	0.179
PROM, *n* (%)	88 (26.5)	34 (25.0)	0.736	18 (27.3)	16 (22.9)	0.552
GDM, *n* (%)	63 (19.0)	27 (19.9)	0.827	13 (19.7)	14 (20.0)	0.965
HDP, *n* (%)	21 (6.3)	8 (5.9)	0.857	3 (4.5)	5 (7.1)	0.780
Pulmonary hemorrhage, *n* (%)	11 (3.3)	26 (19.1)	<0.001	4 (6.1)	22 (31.4)	<0.001
Sepsis, *n* (%)	59 (17.8)	55 (40.4)	<0.001	23 (34.8)	32 (45.7)	0.197
NEC, *n* (%)	8 (2.4)	10 (7.4)	0.012	3 (4.5)	7 (10.0)	0.374
hsPDA, *n* (%)	39 (11.7)	51 (37.5)	<0.001	21 (31.8)	30 (42.9)	0.184
EUGR, *n* (%)	273 (82.2)	116 (85.3)	0.422	55 (83.3)	61 (87.1)	0.531
Grade 3/4 IVH, *n* (%)	6 (1.8)	7 (5.1)	0.092	1 (1.5)	6 (8.6)	0.141
ROP, *n* (%)	233 (70.2)	101 (74.3)	0.38	59 (89.4)	42 (60.0)	<0.001
PS, *n* (%)	325 (97.9)	133 (97.8)	1.000	64 (97)	69 (98.6)	0.959
NIV, *n* (%)	329 (99.1)	127 (93.4)	0.001	66 (100)	61 (87.1)	0.008
IMV, *n* (%)	39 (11.7)	69 (50.7)	<0.001	21 (31.8)	48 (68.6)	<0.001
Duration of IMV, median (*IQR*), days	0 (0–0)	2 (0–15)	<0.001	0 (0–3)	11 (0–22)	<0.001
RBCTs, *n* (%)	159 (47.9)	113 (83.1)	<0.001	53 (80.3)	60 (85.7)	0.400
RBCTs, median (*IQR*), *n*	0 (0–1)	2 (1–3)	<0.001	1 (1–3)	3 (1–4)	0.001
Duration of antibiotic therapy, median (*IQR*), days	16 (10–25)	30 (17–41)	<0.001	26 (17–37)	30 (20–47)	0.102
Postnatal corticosteroids, median (*IQR*), days	1 (0–2)	1 (0–6)	<0.001	1 (0–4)	1 (0–6)	0.759
Breastfeeding, *n* (%)	290 (87.3)	120 (88.2)	0.792	63 (95.5)	57 (81.4)	0.011
FB on day 3, mean ± *SD*	−8.2 ± 3.7	−7.7 ± 3.9	0.238	−8.5 ± 3.8	−7.0 ± 3.9	0.019
FB on day 7, mean ± *SD*	−6.4 ± 4.7	−6.4 ± 5.3	0.979	−7.9 ± 5.4	−4.9 ± 4.8	<0.001
>100 kcal/kg/day by week 1^a^, *n* (%)	53 (16)	12 (8.8)	0.043	9 (13.6)	3 (4.3)	0.055
>120 kcal/kg/day by week 4^a^, *n* (%)	181 (54.5)	37 (27.2)	<0.001	25 (37.9)	12 (17.1)	0.007

BPD, bronchopulmonary dysplasia; GA, gestational age; BW, birth weight; PROM, premature rupture of membranes; GDM, gestational diabetes mellitus; HDP, hypertensive disorders of pregnancy; NEC, necrotizing enterocolitis; hsPDA, hemodynamically significant patent ductus arteriosus; EUGR, extrauterine growth restriction; IVH, intraventricular hemorrhage; ROP, retinopathy of prematurity; PS, pulmonary surfactant; NIV, non-invasive ventilation; IMV, invasive mechanical ventilation; RBCTs, red blood cell transfusions; FB, fluid balance.

^a^ Data are *n* (%) of infants achieving caloric intake: >100 kcal/kg/day by the end of week 1; >120 kcal/kg/day by the end of week 4.

Subgroup analysis revealed no statistically significant differences in either BPD incidence (overall or moderate-to-severe) or duration of IMV among the HFOV, CMV, and combined HFOV + CMV therapy groups (all *p* > 0.05). Regarding non-invasive ventilation use, the CPAP + HFNC combination group demonstrated significantly higher incidence rates of both BPD and moderate-to-severe BPD, along with a longer total duration of non-invasive ventilation therapy, compared to the CPAP group (all *p* < 0.05). Similarly, whole blood transfusion was associated with significantly greater BPD and moderate-to-severe BPD incidence vs. packed red blood cell transfusion (all *p* < 0.05). See Table 3 for details.

### 3.3 Analysis of risk factors for BPD and moderate-to-severe BPD

The potential risk factors and predictors included in the logistic regression models comprised: (1) Demographic factors: GA, BW, sex, multiple pregnancy (vs. singleton pregnancy), cesarean delivery (vs. vaginal), 5-min Apgar score. (2) Maternal factors: maternal age, antenatal corticosteroid use, PROM, GDM, HDP. (3) Complications: pulmonary hemorrhage, sepsis, NEC, hsPDA, EUGR, grade 3/4 IVH (vs. no IVH or grade 1/2 IVH). (4) Treatment-related factors: use of surfactant, duration of IMV, RBCTs frequency and product type (whole blood vs. packed red blood cell), duration of antibiotic therapy, duration of postnatal corticosteroid use, breastfeeding (vs. formula), FB on postnatal days 3 and 7, achievement of caloric intake targets (>100 kcal/kg/day by week 1, >120 kcal/kg/day by week 4).

The analysis revealed that prolonged duration of IMV, increased frequency of RBCTs, extended antibiotic therapy, and the presence of hsPDA were independent risk factors for BPD (all *p* < 0.05). Additionally, prolonged IMV and higher FB on postnatal day 7 were identified as independent risk factors for moderate-to-severe BPD, whereas higher BW was found to be protective against moderate-to-severe BPD (*p* < 0.05), as detailed in Table 5. When the transfusion cohort was stratified by blood product type (whole blood vs. packed red blood cells), multivariate analysis demonstrated that whole blood transfusion was associated with a significantly elevated risk of both BPD and moderate-to-severe BPD compared to packed red blood cell transfusion (*p* < 0.05). Comprehensive results are summarized in - Table 6.

**Table 5:** Multivariate logistic regression analysis of risk factor for BPD and moderate-to-severe BPD.

Variable	Unadjusted OR (95% CI)	*p*	Adjusted OR (95% CI)	*p*
Any BPD (*n* = 468)				
GA	0.51 (0.41–0.62)	<0.001	1.00 (0.70–1.43)	0.998
BW	0.996 (0.995–0.998)	<0.001	1.00 (1.00–1.00)	0.848
Duration of IMV	1.17 (1.12–1.23)	<0.001	1.10 (1.03–1.17)	0.002
RBCTs	2.18 (1.82–2.61)	<0.001	1.61 (1.30–2.01)	< 0.001
Duration of antibiotic therapy	1.07 (1.05–1.09)	<0.001	1.03 (1.01–1.06)	0.011
Duration of postnatal corticosteroids	1.22 (1.13–1.31)	<0.001	0.96 (0.87–1.07)	0.444
5 min Apgar scores ≤7	1.67 (1.1–2.53)	0.017	1.01 (0.59–1.74)	0.968
Pulmonary hemorrhage	6.90 (3.30–14.42)	<0.001	0.94 (0.32–2.77)	0.905
Sepsis	3.14 (2.02–4.89)	<0.001	1.33 (0.71–2.50)	0.370
NEC	3.21 (1.24–8.33)	0.016	0.75 (0.21–2.75)	0.669
hsPDA	4.51 (2.78–7.30)	<0.001	2.27 (1.22–4.20)	0.009
>100 kcal/kg/day by week 1	0.51 (0.26–0.99)	0.046	0.92 (0.41–2.06)	0.836
>120 kcal/kg/day by week 4	0.31 (0.20–0.48)	<0.001	1.13 (0.64–2.02)	0.676
Moderate-to-severe BPD (*n* = 136)				
GA	0.54 (0.38–0.75)	<0.001	1.84 (0.91–3.74)	0.092
BW	0.994 (0.991–0.996)	<0.001	0.993 (0.988–0.998)	0.008
Duration of IMV	1.16 (1.09–1.24)	<0.001	1.16 (1.06–1.27)	0.002
RBCTs	1.38 (1.12–1.70)	0.002	1.15 (0.84–1.57)	0.395
Duration of antibiotic therapy	1.02 (1.00–1.04)	0.039	0.99 (0.96–1.02)	0.517
FB on postnatal day 3	1.11 (1.02–1.22)	0.022	0.85 (0.72–1)	0.051
FB on postnatal day 7	1.13 (1.05–1.21)	0.001	1.19 (1.05–1.34)	0.006
Pulmonary hemorrhage	7.10 (2.30–21.99)	<0.001	3.94 (0.75–20.87)	0.107
Breastfeeding	0.21 (0.06–0.77)	0.019	1.84 (0.28–11.93)	0.522
>100 kcal/kg/day by week 1	0.28 (0.07–1.10)	0.068	0.16 (0.02–1.37)	0.093
>120 kcal/kg/day by week 4	0.34 (0.15–0.75)	0.008	0.97 (0.3–3.15)	0.962

For the model predicting BPD, Hosmer-Lemeshow test *p* = 0.722, Omnibus test *p* < 0.001, Cox and Snell's *R*^2^ = 0.281, Nagelkerke's *R*^2^ = 0.402, AUC = 0.834. For the model predicting moderate-to-severe BPD, Hosmer-Lemeshow test *p* = 0.966, Omnibus test *p* < 0.001, Cox and Snell's *R*^2^ = 0.413, Nagelkerke's *R*^2^ = 0.551, AUC = 0.889. BPD, bronchopulmonary dysplasia; GA, gestational age; BW, birth weight; IMV, invasive mechanical ventilation; RBCTs, red blood cell transfusions; NEC, necrotizing enterocolitis; hsPDA, hemodynamically significant patent ductus arteriosus; FB, fluid balance.

**Table 6:** Multivariate logistic regression analysis of risk factor for BPD and moderate-to-severe BPD in transfusion group.

Variable	Unadjusted OR (95% CI)	*p*	Adjusted OR (95% CI)	*p*
Any BPD (*n* = 272)				
GA	0.51 (0.41–0.62)	<0.001	1.04 (0.68–1.57)	0.868
BW	0.996 (0.995–0.998)	<0.001	1.00 (1.00–1.00)	0.877
Duration of IMV	1.17 (1.12–1.23)	<0.001	1.07 (1.00–1.14)	0.038
RBCTs	2.18 (1.82–2.61)	<0.001	1.20 (0.88–1.64)	0.247
Duration of antibiotic therapy	1.07 (1.05–1.09)	<0.001	1.03 (1.00–1.06)	0.029
Duration of postnatal corticosteroids	1.22 (1.13–1.31)	<0.001	1.08 (0.94–1.24)	0.297
5 min Apgar scores ≤7	1.67 (1.1–2.53)	0.017	1.39 (0.71–2.70)	0.336
Pulmonary hemorrhage	6.90 (3.30–14.42)	<0.001	0.89 (0.29–2.68)	0.835
Sepsis	3.14 (2.02–4.89)	<0.001	1.17 (0.58–2.39)	0.661
NEC	3.21 (1.24–8.33)	0.016	0.79 (0.21–3.01)	0.725
hsPDA	4.51 (2.78–7.30)	<0.001	2.16 (1.04–4.46)	0.038
>100 kcal/kg/day by week 1	0.51 (0.26–0.99)	0.046	0.79 (0.27–2.35)	0.670
>120 kcal/kg/day by week 4	0.31 (0.20–0.48)	<0.001	1.23 (0.60–2.51)	0.578
Whole blood	4.02 (2.39–6.75)	<0.001	4.48 (1.92–10.43)	<0.001
Moderate-to-severe BPD (*n* = 113)				
GA	0.54 (0.38–0.75)	<0.001	1.44 (0.66–3.14)	0.366
BW	0.994 (0.991–0.996)	<0.001	0.994 (0.988–0.999)	0.029
Duration of IMV	1.16 (1.09–1.24)	<0.001	1.11 (1.01–1.21)	0.026
RBCTs	1.38 (1.12–1.70)	0.002	1.06 (0.69–1.62)	0.792
Duration of antibiotic therapy	1.02 (1.00–1.04)	0.039	1.01 (0.98–1.05)	0.550
FB on postnatal day 3	1.11 (1.02–1.22)	0.022	0.87 (0.72–1.06)	0.159
FB on postnatal day 7	1.13 (1.05–1.21)	0.001	1.19 (1.03–1.37)	0.017
Pulmonary hemorrhage	7.10 (2.30–21.99)	<0.001	4.53 (0.77–26.73)	0.095
Breastfeeding	0.21 (0.06–0.77)	0.019	0.88 (0.12–6.62)	0.902
>100 kcal/kg/day by week 1	0.28 (0.07–1.10)	0.068	0.06 (0–1.78)	0.102
>120 kcal/kg/day by week 4	0.34 (0.15–0.75)	0.008	1.37 (0.34–5.49)	0.658
Whole blood	2.82 (1.31–6.07)	0.008	4.81 (1.24–18.63)	0.023

For the model predicting BPD, Hosmer-Lemeshow test *p* = 0.010, Omnibus test *p* < 0.001, Cox and Snell's *R*^2^ = 0.303, Nagelkerke's *R*^2^ = 0.408, AUC = 0.830. For the model predicting moderate-to-severe BPD, Hosmer-Lemeshow test *p* = 0.544, Omnibus test *p* < 0.001, Cox and Snell's *R*^2^ = 0.434, Nagelkerke's *R*^2^ = 0.580, AUC = 0.893. BPD, bronchopulmonary dysplasia; GA, gestational age; BW, birth weight; IMV, invasive mechanical ventilation; RBCTs, red blood cell transfusions; NEC, necrotizing enterocolitis; hsPDA, hemodynamically significant patent ductus arteriosus; FB, fluid balance.

### 3.4 Thresholds of duration of IMV and FB on postnatal day 7 in predicting moderate-to-severe BPD

**Figure 1:**
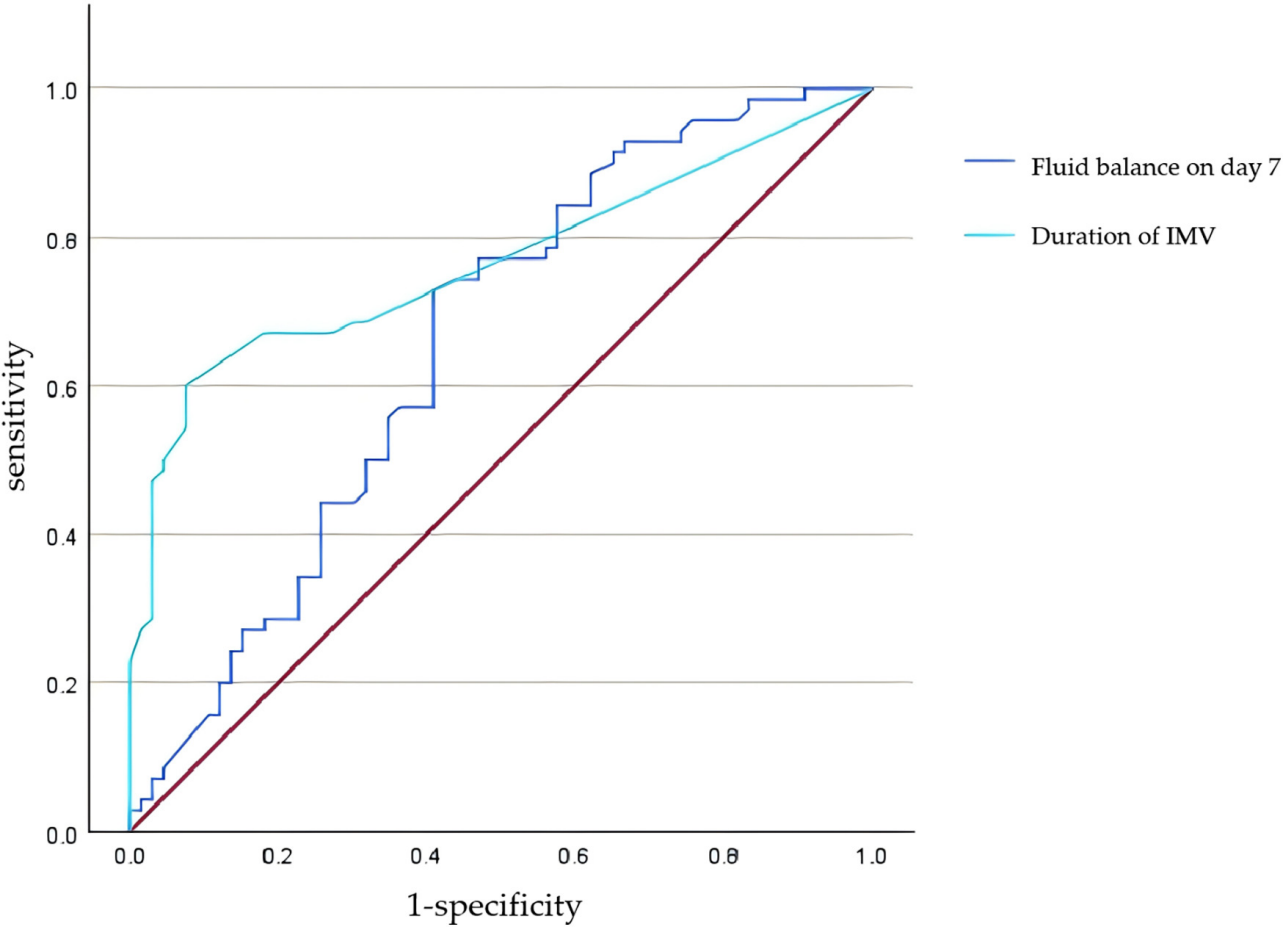
ROC curve for predicting moderate-to-severe BPD by duration of IMV and fluid balance on postnatal day 7.

ROC curves were plotted to evaluate the predictive value of the duration of IMV and FB on postnatal day 7 for moderate-to-severe BPD. For IMV, the optimal cut-off value was 6.5 days, yielding an area under the curve (AUC) of 0.77 (95% CI: 0.69–0. 84, *p* < 0.0001), with sensitivity and specificity of 60.0% and 92.4%, respectively. For FB on postnatal day 7, a cut-off value of −7.2 demonstrated an AUC of 0.66 (95% CI: 0.56–0.75, *p* = 0.001), with sensitivity and specificity of 72.9% and 59.1%, respectively. See Figure 1 and Table 7 for additional details.

**Table 7:** Diagnostic performance of IMV and FB on postnatal Day 7 at various cutoff values.

Variable	Cutoff Value	Sensitivity	Specificity	PPV	NPV	LR+	LR−
IMV	0.5	0.686	0.682	0.480	0.835	2.157	0.460
	6.5	0.600	0.924	0.772	0.844	7.895	0.433
	22.5	0.229	1.000	1.000	0.752	<0.0001	0.771
FB on postnatal day 7	−9.9	0.857	0.379	0.594	0.714	1.380	0.377
	−7.2	0.729	0.591	0.654	0.672	1.781	0.459
	−2.6	0.286	0.788	0.588	0.510	1.347	0.907

IMV, invasive mechanical ventilation; FB, fluid balance; PPV, positive predictive value; NPV, negative predictive value; LR+, positive likelihood ratio; LR−, negative likelihood ratio.

## 4 Discussion

The incidence of BPD has shown a rising trend in recent years. In the United States, the incidence of BPD among infants with a GA <29 weeks ranged from 44.7% to 49.8% between 2008 and 2018 (1), while in Sweden, the incidence among infants with a GA <27 weeks was 65% to 68% between 2004 and 2016 (2). Data from 68 neonatal centers in China showed that the incidence of BPD in EPIs increased from 20.8% in 2010 to 40.7% in 2019 (3). A similar trend was observed at our center, where the BPD incidence rose from 24.2% during 2012–2019 to 34.5% in 2020–2024. This increase is likely associated with a higher proportion of preterm infants born at lower gestational ages. Both lower GA and lower BW are established as the strongest predictors of BPD (12), with disease incidence and severity exhibiting an inverse relationship to these factors. Our multivariate analysis confirmed increased BW as a protective factor against moderate-to-severe BPD, although a significant relationship between GA and BPD was not established. The overall BPD incidence at our center was lower than rates reported in developed countries (1, 2). This discrepancy may be explained by the predominance of infants born at 26 and 27 weeks GA in our cohort (constituting 74.6%), and a significant proportion of infants born at GA <26 weeks either died before 36 weeks PMA or experienced care withdrawal due to socioeconomic challenges. Notably, infants born at <24 weeks GA were particularly underrepresented (only 1.5%), limiting our ability to accurately reflect the true incidence within this highest-risk GA subgroup.

Multivariate analysis in this study identified prolonged IMV as an independent risk factor for both BPD and moderate-to-severe BPD, consistent with previous studies (13, 14). Ventilation-induced lung injury (VILI) is a known risk factor for BPD (15), with mechanisms including volutrauma, barotrauma, atelectrauma, oxygen toxicity, and biotrauma, which interact with patient-specific factors such as immature lungs, surfactant deficiency, non-uniform lung disease, and pulmonary inflammation to exacerbate lung injury. The optimal strategy to mitigate VILI and BPD risk is the use of non-invasive respiratory support. The American Academy of Pediatrics guidelines (16) recommend early application of continuous positive airway pressure at or soon after birth with subsequent selective surfactant therapy. However, avoiding IMV at all costs may expose some preterm infants to prolonged episodes of apnea or worsening respiratory distress syndrome, ultimately necessitating rescue invasive ventilation. Lung-protective ventilation strategies, such as volume-targeted ventilation, have been shown in a meta-analysis (17) to significantly reduce the composite outcome of death or BPD at 36 weeks PMA compared to pressure-controlled ventilation. This subgroup analysis revealed no significant differences in BPD incidence (overall or moderate-to-severe) or IMV duration among HFOV, CMV, or HFOV + CMV groups. In contrast, the CPAP + HFNC group exhibited significantly higher BPD and moderate-to-severe BPD rates and longer NIV duration vs. CPAP alone. This observation is likely attributable to the relatively limited respiratory support pressure provided by HFNC. As a well-established non-invasive modality, CPAP delivers more reliable alveolar recruitment, suggesting it remains a preferable clinical option over HFNC supplementation in this context.

Previous studies have identified RBCTs as a risk factor for BPD (18), a finding corroborated by our study. The incidence of BPD increased with the number of transfusions, potentially explained by transfusion-related immunomodulation (TRIM), which may play a significant role in transfusion-associated diseases (19). Crawford found that early and repeated transfusions in very preterm infants led to changes in pro-inflammatory cytokines and endothelial activation markers, suggesting that TRIM may occur within the first few days of life (20). Iron and other inflammatory mediators in blood products can promote free radical generation, infection, and fibrosis (21), with oxidative stress and inflammation being key mechanisms in BPD pathogenesis. Additionally, transfusion-related acute lung injury (TRALI) and transfusion-associated circulatory overload (TACO) are the most common cardiopulmonary complications of transfusions (22), causing neutrophil activation, endothelial damage, protein leakage, increased hydrostatic pressure, or fluid extravasation, all of which contribute to pulmonary edema and ultimately lead to lung injury. Stainsby (23) observed that the incidence of adverse transfusion reactions increases progressively among adults, children, and infants, suggesting that neonates may be more susceptible to such adverse reactions. Bedford (24) found that preterm infants receiving multiple transfusions of non-leukoreduced whole blood developed anti-human leukocyte antigen antibodies, while those receiving leukoreduced blood did not. In our center, for infants requiring multiple transfusions within a short period, blood from a single donor is prioritized to minimize exposure to multiple donors. Multivariate analysis stratified by blood product type (whole blood vs. packed red blood cells) revealed that whole blood transfusion was associated with a higher risk of BPD and moderate-to-severe BPD compared to packed red blood cells. Since the whole blood transfused in our center was not leukoreduced, this suggests that TRIM may be related to the increased risk of BPD. Crawford (25) suggested that transfusing washed red blood cells might mitigate this response, and a prospective randomized controlled trial (26) is currently underway to evaluate the impact of washed vs. unwashed red blood cells on morbidity and mortality in EPIs. Recent guidelines (27), based on extensive randomized clinical trials, indicate that low-threshold transfusion does not increase short or long-term adverse outcomes, including BPD, and recommend restrictive transfusion strategy.

Previous studies have demonstrated that fluid overload or higher FB increases the risk of BPD (8, 28). Preterm infants typically undergo physiological diuresis postnatally, leading to a negative FB. However, fluid overload may induce pulmonary edema and inflammation, impairing the normal development of alveoli and pulmonary vasculature. Additionally, fluid overload can prolong the patency of PDA (29), while acute kidney injury (30) or medications such as indomethacin (31) may further compromise diuretic processes, exacerbating fluid retention and thereby contributing to lung injury and BPD progression. However, Diderholm (32), based on fluid intake, suggested that early fluid restriction did not reduce BPD incidence, a discrepancy potentially attributable to variations in study protocols. This study demonstrated that higher FB on postnatal day 7 was an independent risk factor for moderate-to-severe BPD, which is consistent with previous findings (8). Maintaining the FB on postnatal day 7 below −7.2 may be an effective preventive measure against BPD. However, since FB was assessed solely via weight changes rather than direct measurement of fluid intake or output, clinical practice should integrate GA, weight trends, and fluid intake and output data to refine individualized fluid management strategies. Prospective studies are warranted to validate the impact of fluid management on BPD outcomes.

Due to the immature immune systems of preterm infants, invasive procedures, and the use of medical devices, antibiotics are among the most commonly prescribed medications in the NICU (33). However, excessive antibiotic exposure may lead to increased antibiotic resistance and alter the gut microbiome (34). Previous studies have linked excessive antibiotic exposure to an elevated risk of adverse outcomes, including BPD, in preterm infants (35), a finding supported by our study. The gut-lung axis hypothesis provides a potential mechanism, suggesting that gut dysbiosis may disrupt the intestinal barrier, triggering inflammation, metabolic disturbances, and malnutrition, which in turn exacerbate pulmonary inflammation and injury, increasing the risk of BPD (36). The imbalance in the airway microbiota, influenced by factors such as antibiotic therapy and mechanical ventilation, is linked to the occurrence of BPD (37), while the gut and airway microbiomes interact bidirectionally through the gut-lung axis, contributing to abnormal inflammatory responses that play a critical role in the pathogenesis of BPD (37). Therefore, reducing unnecessary antibiotic exposure is critical. Fischer (38) demonstrated that a quality improvement project implementing standardized antibiotic therapy guidelines successfully reduced antibiotic utilization by 24.8%, with sustained effects over three years, highlighting the feasibility of reducing antibiotic exposure through education, ongoing feedback, and monitoring.

This study confirmed that hsPDA is a significant risk factor for BPD, consistent with existing research (39, 40). The persistent left-to-right shunt in hsPDA increases pulmonary blood flow, leading to pulmonary edema, reduced lung compliance (41), and even pulmonary hemorrhage (42), thereby increasing the need for mechanical ventilation and oxygen, which exacerbates the risk of VILI. Increased pulmonary blood flow also triggers pulmonary neutrophil margination and activation (43), further intensifying lung inflammation and contributing to BPD development. While it is logical to assume that closing the PDA would reduce BPD incidence, studies have shown that pharmacological or surgical interventions do not improve BPD rates and may even increase them (31, 44, 45). Huang (46) found that ibuprofen may inhibit vascular growth factor levels in preterm infants, potentially explaining the increased BPD risk. The optimal management of PDA remains controversial, indicating that its role in BPD pathogenesis may be more complex than previously thought.

To our knowledge, this is the largest single-center retrospective analysis of EPIs in China, providing critical insights through its large-scale longitudinal design. Multivariable logistic regression models were employed to adjust for confounding factors, and the independent predictive value of FB was rigorously validated through statistical analyses. Importantly, as a highly modifiable clinical parameter, FB may provide a direct intervention target for optimizing fluid management in preterm infants. Additionally, this study is the first to demonstrate that whole-blood transfusion was associated with a higher risk of BPD compared to traditional packed red blood cell transfusion. This study has several inherent limitations: (1) its retrospective design and single-center scope may introduce selection bias and limit generalizability, compounded by insufficient consideration of confounders such as socioeconomic status, treatment strategy variations, and evolving clinical protocols; (2) due to incomplete data on maternal infections, this factor was not included in the analysis; (3) only short-term outcomes were analyzed, and long-term follow-up is needed to evaluate the relationship between BPD and growth, cardiopulmonary, and neurodevelopmental outcomes.

## 5 Conclusions

In summary, BPD is a multifactorial condition influenced by synergistic interactions of modifiable and non-modifiable risk factors. Independent risk factors for BPD include prolonged IMV, extended antibiotic exposure, frequent RBCTs, whole blood transfusion (compared to packed RBCTs), and hsPDA. For moderate-to-severe BPD, disease severity was independently associated with prolonged IMV, whole-blood transfusion, and higher FB on postnatal day 7, while higher birth weight was identified as a protective factor. Clinically, implementing stratified prevention strategies is critical: shortening duration of IMV through non-invasive ventilation support or lung-protective strategies (e.g., volume-targeted ventilation), maintaining an appropriate negative FB early, adopting restrictive transfusion protocols, and standardizing antibiotic stewardship. Future multicenter prospective studies with expanded demographic data collection and enhanced control of confounding variables are warranted to improve generalizability and analytical rigor, ultimately enabling precise prevention and management of bronchopulmonary dysplasia in very preterm infants.

## Data Availability

The raw data supporting the conclusions of this article will be made available by the authors, without undue reservation.
